# Aging Affects K_V_7 Channels and Perivascular Adipose Tissue-Mediated Vascular Tone

**DOI:** 10.3389/fphys.2021.749709

**Published:** 2021-11-26

**Authors:** Yibin Wang, Fatima Yildiz, Andrey Struve, Mario Kassmann, Lajos Markó, May-Britt Köhler, Friedrich C. Luft, Maik Gollasch, Dmitry Tsvetkov

**Affiliations:** ^1^Charité Medical Faculty, Experimental and Clinical Research Center (ECRC), Max Delbrück Center for Molecular Medicine (MDC), Berlin, Germany; ^2^Faculty of Medicine, Istanbul Medeniyet University, Istanbul, Turkey; ^3^Department of Ear, Throat and Nose Diseases, I.M. Sechenov First Moscow State Medical University, Moscow, Russia; ^4^Department of Internal Medicine and Geriatrics, University Medicine Greifswald, Greifswald, Germany

**Keywords:** aging, K_V_7 channels, perivascular adipose tissue, transcriptome, RNA sequencing

## Abstract

Aging is an independent risk factor for hypertension, cardiovascular morbidity, and mortality. However, detailed mechanisms linking aging to cardiovascular disease are unclear. We studied the aging effects on the role of perivascular adipose tissue and downstream vasoconstriction targets, voltage-dependent K_V_7 channels, and their pharmacological modulators (flupirtine, retigabine, QO58, and QO58-lysine) in a murine model. We assessed vascular function of young and old mesenteric arteries *in vitro* using wire myography and membrane potential measurements with sharp electrodes. We also performed bulk RNA sequencing and quantitative reverse transcription-polymerase chain reaction tests in mesenteric arteries and perivascular adipose tissue to elucidate molecular underpinnings of age-related phenotypes. Results revealed impaired perivascular adipose tissue-mediated control of vascular tone particularly *via* K_V_7.3–5 channels with increased age through metabolic and inflammatory processes and release of perivascular adipose tissue-derived relaxation factors. Moreover, QO58 was identified as novel pharmacological vasodilator to activate XE991-sensitive KCNQ channels in old mesenteric arteries. Our data suggest that targeting inflammation and metabolism in perivascular adipose tissue could represent novel approaches to restore vascular function during aging. Furthermore, K_V_7.3–5 channels represent a promising target in cardiovascular aging.

## Introduction

Hypertension is the leading risk factor of death worldwide, especially for persons aged 50–74years and 75years and older (Collaborators, 2020). Human life expectancy is increasing steadily, which will further amplify age-related effects (Vollset et al., 2020). Rigorous research is necessary to address these challenges. Despite remarkable progress in understanding molecular biology of aging, detailed mechanisms linking aging to cardiovascular disease are still unclear (North and Sinclair, 2012). Mice are a utilitarian model to investigate age-related effects (Dupont et al., 2016). Our past studies have shed light on the regulation of vasculature tone by perivascular adipose tissue (PVAT; Gollasch, 2017). Fatty tissue surrounding blood vessels is now recognized as an integral endocrine/paracrine organ. In addition to the endothelium, PVAT releases vasoactive compounds to cause relaxation of blood vessels known as the anticontractile effect of PVAT (Lohn et al., 2002). PVAT relaxation factors (PVATRFs) have been proposed and such factors could be pivotal in aging. Our earlier work suggests that PVAT paracrine effects are caused by opening of potassium (K^+^) channels in vascular smooth muscle cells (Verlohren et al., 2004). The KCNQ-type, K_V_7 channels represent the most likely candidates as largely supported by studies with XE991, a highly effective blocker of these channels (Schleifenbaum et al., 2010; Tsvetkov et al., 2017). In fact, the K_V_7 family represents a new target for hypertension treatment (Schleifenbaum et al., 2010; Jepps et al., 2011; Mani et al., 2016). K_V_7 channel function determines sensitivity to key regulators of coronary tone in diabetes, which expands therapeutic potential even further (Morales-Cano et al., 2015; Barrese et al., 2018). However, toxicity issues of currently available K_V_7 channel modulators, such as retigabine and flupirtine, have hampered drug development directed at this target (FDA, 2013; European Medicines Agency, 2018). Pyrazolo[1,5-a]pyrimidin-7(4H)-one compounds (e.g., QO58) have been identified as novel KCNQ channel openers, which can cause remarkable leftward shifts of voltage-dependent activation of K_V_7 channels (Jia et al., 2011). Newly emerging RNA sequencing technologies coupled with established techniques could enable researching these new compounds (Tabula Muris, 2020). We hypothesize that age could attenuate the effects of PVAT as mediated by K_V_7 channels. We employed the established flupirtine and retigabine, as well as novel compounds (QO58 and QO58-lysine) as K_V_7 channel activators in isolated mesenteric arteries from young and old mice.

## Materials and Methods

### Mouse Model

We used young (11–18weeks old), 12-month old (50–54weeks), 16-month old (66–69weeks), and 24-month old (105–106weeks) male wild-type mice C57BL/6N. Animal care followed American Physiological Society guidelines, and local authorities (Landesamt für Gesundheit und Soziales Berlin, LAGeSo) approved all protocols. Mice were housed in individually ventilated cages under standardized conditions with an artificial 12-h dark–light cycle with free access to water and food. Animals were randomly assigned to the experimental procedures in accordance with the German legislation on protection of animals.

### Wire Myography

Mesenteric arteries were isolated after sacrifice with isoflurane anesthesia, as previously described (Tsvetkov et al., 2017). Then, blood vessels were quickly transferred to cold (4°C), oxygenated (95% O_2_/5% CO_2_) physiological salt solution (PSS) containing (in mmol/L) 119 NaCl, 4.7 KCl, 1.2 KH_2_PO_4_, 25 NaHCO_3_, 1.2 Mg_2_SO_4_, 11.1 glucose, and 1.6 CaCl_2_. We dissected the vessels into 2mm rings whereby perivascular fat and connective tissue were either intact [(+) PVAT] or removed [(−) PVAT rings]. Each ring was placed between two stainless steel wires (diameter 0.0394mm) in a 5-ml organ bath of a Mulvany Small Vessel Myograph (DMT 610M; Danish Myo Technology, Denmark). The organ bath was filled with PSS. Continuously oxygenated bath solution with a gas mixture of 95% O_2_ and 5% CO_2_ was kept at 37°C (pH 7.4). To obtain the passive diameter of the vessel at 100mm Hg, a DMT normalization procedure was performed. The mesenteric artery rings were placed under a tension equivalent to that generated at 0.9 times the diameter of the vessel at 100mm Hg by stepwise distending the vessel using LabChart DMT Normalization module. The software Chart5 (AD Instruments Ltd. Spechbach, Germany) was used for data acquisition and display. After 60-min incubation, arteries were precontracted either with isotonic external 60mm KCl or 1–3μm phenylephrine (PE), or methoxamine (ME) until a stable resting tension was acquired. The composition of 60mM KCl (in mmol/L) was 63.7 NaCl, 60 KCl, 1.2 KH_2_PO_4_, 25 NaHCO_3_, 1.2 Mg_2_SO_4_, 11.1 glucose, and 1.6 CaCl_2_. Drugs were added to the bath solution if not indicated otherwise. Tension is expressed as a percentage of the steady-state tension (100%) obtained with isotonic external 60mm KCl or agonist (e.g., PE and ME).

### Membrane Potential Recordings

Intracellular recordings of membrane potential in smooth muscle cells of intact mesenteric arteries were made using microelectrodes pulled from aluminosilicate glass and filled with 3M KCl as previously described (Zavaritskaya et al., 2020). An amplifier (DUO 773, World Precision Instruments) was used to record the membrane potential. We used a micromanipulator (UMP, Sensapex) to make impalements from the vessel’s adventitial side. The following criteria for acceptance of membrane potential recordings were used: (1) an abrupt change in membrane potential upon cell penetration; (2) a constant electrode resistance when compared before, during, and after the measurement; (3) a stable reading of the membrane potential lasting longer than 1min; and (4) no change in the baseline when the electrode was removed.

### Histology

Formalin-fixed, paraffin-embedded, 4-μm-thick sections were hematoxylin- and eosin-stained using standard protocols. Sections were scanned using the Slide Scanner Pannoramic MIDI (3DHistech Ltd., Hungary) with the objective plan-apochromat 20x (ZEISS, Germany). Forty randomly chosen fat cells were measured and analyzed using CaseViewer (3DHistech Ltd., Hungary) software, and mean perimeter and area were calculated. Immunohistochemical staining of Ly-6B.2-positive cells was performed on 4-μm-thick formalin-fixed, paraffin-embedded sections. Antigen retrieval was performed by incubating sections for 10min at 37°C in a trypsin solution (Sigma). After cooling down, non-specific binding sites were blocked with 10% normal donkey serum for 30min following incubation with rat anti-mouse Ly-6B.2 monoclonal antibody (MCA771G, AbD Serotec, clone 7/4, dilution: 1:300) overnight at 4°C in a humid chamber. For fluorescence visualization of bound primary antibody, sections were further incubated with Cy3-conjugated secondary antibody for 1h in a humid chamber at room temperature. Specimens were analyzed using a Zeiss Axioplan-2 imaging microscope with AxioVision 4.8 software (Zeiss, Jena, Germany). The investigator had no knowledge of the treatment group assignment. Ly-6B.2-positive cells were counted through the whole section using 400X magnification; mean of two sections are presented.

### Quantitative Real-Time PCR

Total RNA was isolated from young, 12-, 16-, and 24-month-old mice mesenteric arteries (first branches) by using the RNeasy RNA isolation kit (Qiagen, Germantown, MD) according to the manufacturer’s instruction. Isolated RNA concentration was measured, and RNA quality was tested by NanoDrop-1000 spectrophotometer (Thermo Fisher Scientific, Vernon Hills, IL). Two micrograms of RNA was used for cDNA transcription (Applied Biosystems, Foster City, CA). Experiments were run on an Applied Biosystems 7500 Fast Real-Time PCR System (Life Technologies Corporation, Carlsbad, CA, United States). Primers were designed using Primer 3 software on different exons to exclude any DNA contamination. Specificity of amplified products was validated *in silico* (blast) and empirically with gel electrophoresis and analysis of melt curves. Primers were synthesized by BioTez (Berlin, Germany); the sequences are provided below. The cycling conditions were the following: initial activation at 95°C for 10min, followed by 40cycles at 95°C for 15s and 60°C for 1min. Samples and negative controls were run in parallel. Quantitative analysis of target mRNA expression was performed with quantitative real-time PCR using the relative standard curve method. The expression level of the target genes was normalized by the expression of *18s*. Under our experimental conditions, expression of *18s* as a reference gene did not differ between young and old mice tissues. The fold change in gene expression between young and old mice was calculated using 2^ΔΔCt^ method. The following primers were used:

*18s*: F: 5′-ACATCCAAGGAAGGCAGCAG-3′;

R: 5′-TTTTCGTCACTACCTCCCCG-3′.

*Kcnq1*: F: 5′-AGCAGTATGCCGCTCTGG-3′;

R: 5′-AGATGCCCACGTACTTGCTG-3′.

*Kcnq3*: F: 5′-CAGTATTCGGCCGGACATCT-3′;

R: 5′-GAGACTGCTGGGATGGGTAG-3′.

*Kcnq4*: F: 5′-CACTTTGAGAAGCGCAGGAT-3′;

R: 5′-CCAGGTGGCTGTCAAATAGG-3′.

*Kcnq5*: F: 5′-CCTCACTACGGCTCAAGAGT-3′;

R: 5′-TTAAGTGGTGGGGTGAGGTC-3′.

### RNA Sequencing

Following Agilent 2100 bioanalyzer quality control, RNA-seq was performed using Illumina Genome Analyzer Novaseq 6000 platform. NEB Next^®^ Ultra^™^ RNA Library Prep Kit was used for library preparation. Sequence quality estimations, GC content, nucleotide distribution, and read duplication levels were determined for the samples using fastp-0.12.2 software. The reads were mapped to the reference mouse genome (ensembl_mus_musculus_grcm38_p6_gca_000001635_8). HISAT2 was selected to map the filtered sequenced reads to the reference genome. The uniquely mapped read data output was processed using custom scripts in R software (version 3.5.1) and then normalized using the FeatureCounts package v1.5.0-p3 version. Differential expression analysis was performed using the DESeq2 R package version v1.20.0 (Anders and Huber, 2010). We used clusterProfiler for enrichment analysis, including GO Enrichment, DO Enrichment, Kyoto Encyclopedia of Genes and Genomes (KEGG), and Reactome database Enrichment (Yu et al., 2012). Heat map was generated based on fragments per kilobase per million mapped fragments values using Morpheus software.^1^

### Materials

All salts and other chemicals were purchased from Sigma-Aldrich (Germany) or Merck (Germany). Using DMSO or PSS, drugs were freshly dissolved on the day of each experiment accordingly to the material sheet. Maximal DMSO concentration after application did not exceed 0.5%. Following concentration of drugs was used: phenylephrine (Sigma-Aldrich) and methoxamine (Sigma-Aldrich) ranged from 0.01 to 100μm; retigabine (Valeant Research North America), flupirtine (Tocris), QO58 (Tocris), QO58-lysine from 0.01 to 30μm; 3μm XE991 (Tocris).

### Statistics

Data present mean±SEM. We calculated EC_50_ values using a Hill equation: T=(B_0_ – Be)/(1+([D]/EC_50_)^n^)+Be, where T is the tension in response to the drug (D); Be is the maximum response induced by the drug; B_0_, is a constant; EC_50_ is the concentration of the drug that elicits a half-maximal response.

For curve fittings using non-linear regression, GraphPad 8.0.1 (Software, La Jolla California United States) software was used. Statistical significance was determined by Mann–Whitney test or nonparametric ANOVA (Kruskal-Wallis test). Extra sum-of-squares F test was performed for comparison of concentration-response curves. Values of *p*<0.05 were considered statistically significant. n represents the number of arteries; N represents the number of mice tested. Figures were made using Coreldraw Graphics Suite 2020 (Ottawa, Canada).

## Results

### Aging Impairs PVAT-Mediated Control of Vascular Tone

**Figure 1:**
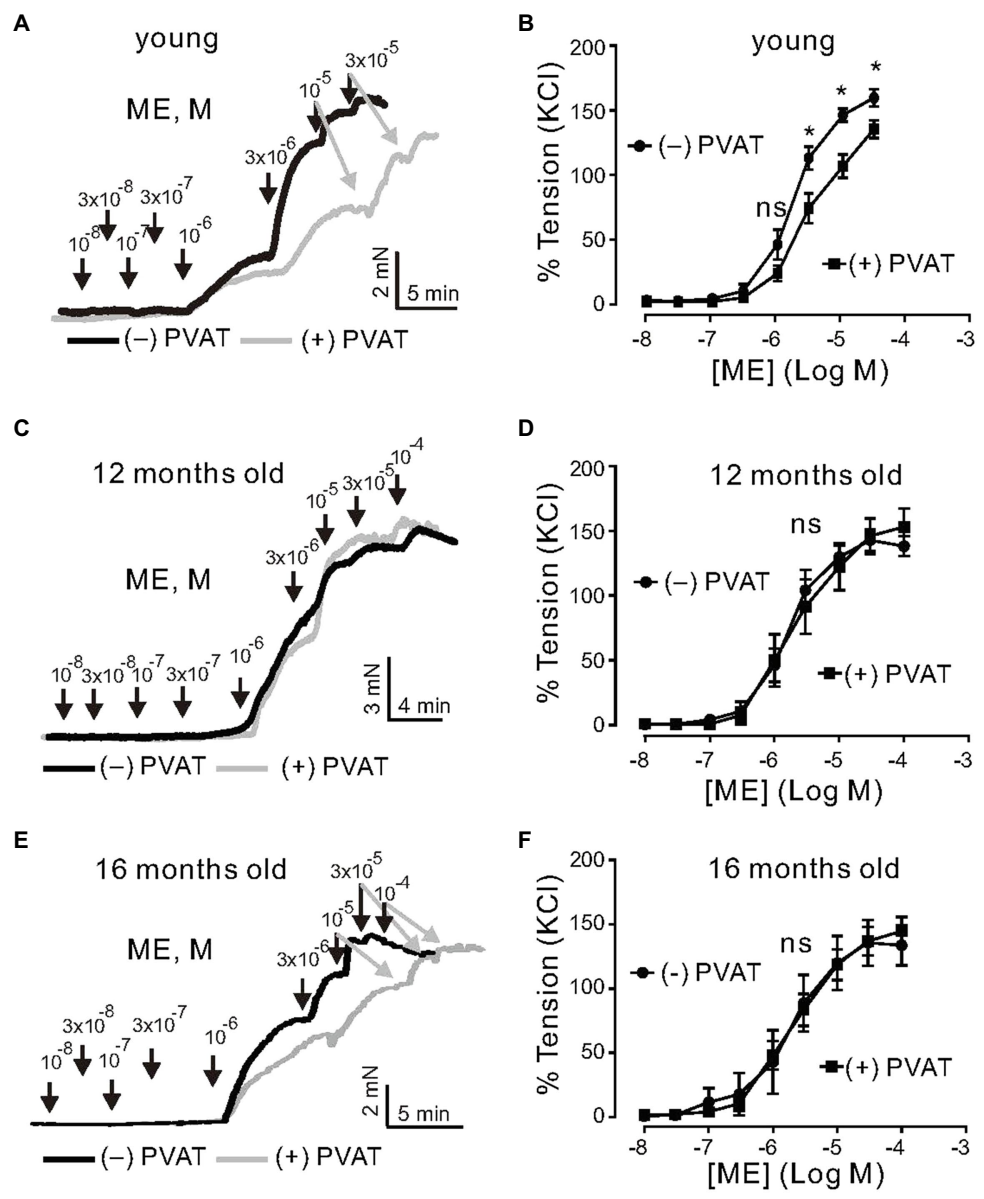
Effects of aging on regulation of arterial tone by a1-agonists Methoxamine (ME) and perivascular adipose tissue (PVAT). **(A)** Original traces showing a1-agonist-induced contractions in (−) PVAT and (+) PVAT mesenteric artery rings isolated from young mice. **(B)** Concentration-response relationships for a1-agonist-induced contractions in (−) PVAT (*n*=10, *N*=5) or (+) PVAT (*n*=10, *N*=4) mesenteric arteries from young animals. **(C)** Original traces showing aging effects on a1-agonist-induced contractions in (−) PVAT and (+) PVAT mesenteric artery rings isolated from 12-month-old mice. **(D)** Concentration-response relationships for a1-agonist-induced contractions of (+) PVAT (*n*=6, *N*=2) and (−) PVAT (*n*=9, *N*=2) artery rings isolated from 12-month-old mice. **(E)** Original traces showing aging effects on a1-agonist-induced contractions in (−) PVAT and (+) PVAT mesenteric artery rings isolated from 16-month-old mice. **(F)** Cumulative concentration-response relationships to a1-agonist in (−) PVAT (*n*=7, *N*=3) and (+) PVAT (*n*=9, *N*=3) mesenteric arteries in 16-month-old mice. ^*^p<0.05. Two-way ANOVA followed by Bonferroni *post hoc* test. Data are mean and SEM.

First, we examined the role of aging in the anticontractile effects of PVAT. Isolated mesenteric arteries were contracted by alpha1 adrenoceptor (alpha1-AR) stimulation with methoxamine (ME). To test whether or not PVAT regulation on the arterial tone is impaired with aging, we performed a series of experiments using arteries from young (3months old), 12-, and 16-month-old mice (Figure 1). Arteries were prepared either with (+) PVAT or without (−) PVAT. Mesenteric artery rings of young mice displayed strong anticontractile effects of PVAT, namely, the concentration-response curve for vasocontractions of (+) PVAT rings by ME was shifted to the right, compared to (−) PVAT rings (Figures 1A,B). In contrast, (−) PVAT artery rings from 1-year-old mice displayed contractions in response to alpha1-AR agonist similar to (+) PVAT rings (Figures 1C,D). To substantiate the results, we performed similar experiments using artery rings isolated from 16-month-old mice. Alpha1-AR agonist-induced contractions were similar between (−) PVAT rings and (+) PVAT rings (Figures 1E,F). Together, the results suggest that the anticontractile effects of PVAT are impaired in aging.

### K_V_7 Channel Function in PVAT Is Affected by Age

**Figure 2:**
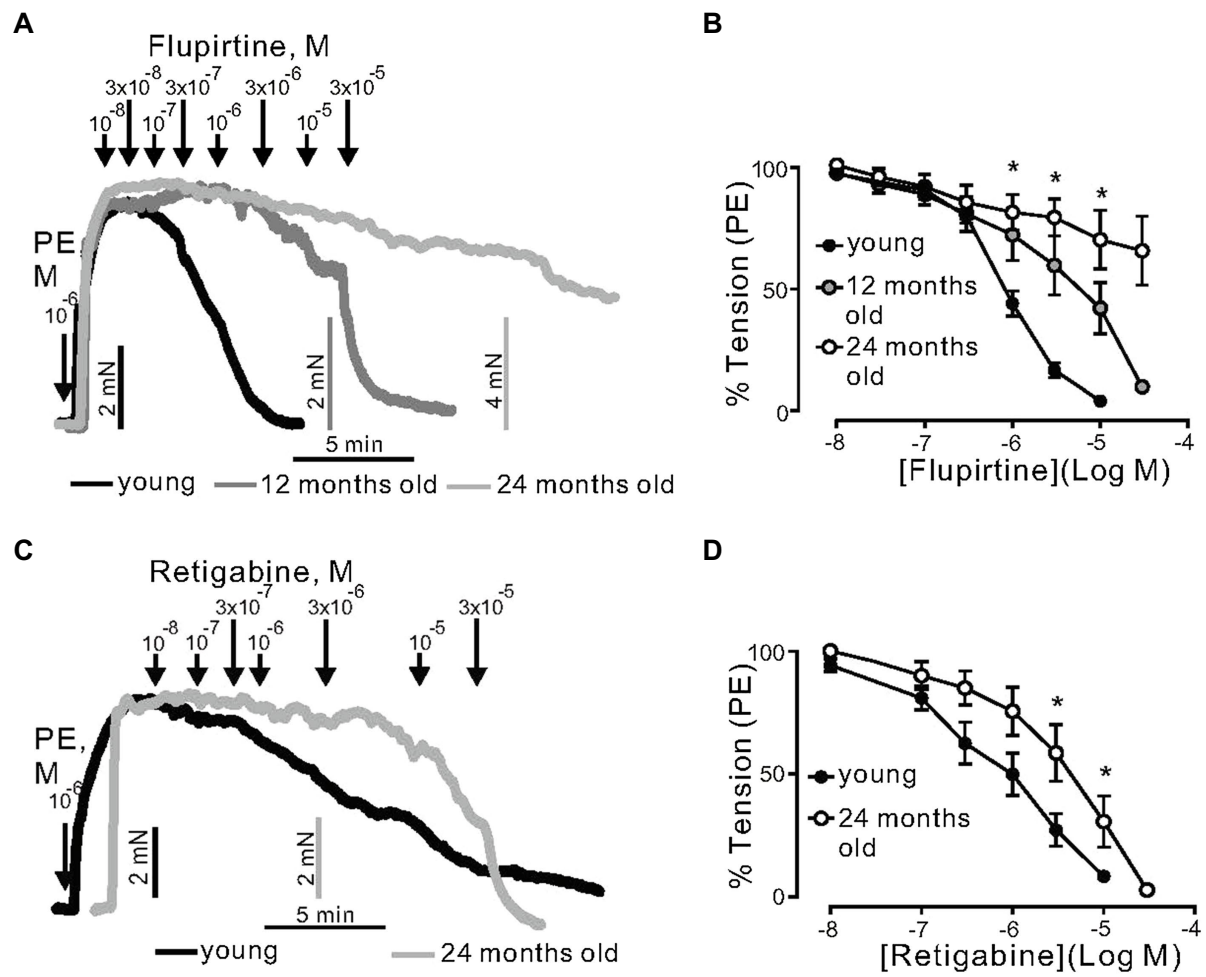
Relaxation of (−) PVAT mesenteric artery rings by KCNQ channels activators flupirtine and retigabine. **(A)** Representative traces showing the relaxation induced by 0.01–30μm flupirtine in rings isolated from young, 12-months, and 24-month-old mice. Mesenteric arteries were precontracted by 1μm phenylephrine (PE). **(B)** Concentration-response relationships for flupirtine-induced relaxation in mesenteric arteries in young (*n*=12, *N*=3), 12-month-old (*n*=10, *N*=4), and 24-month-old (*n*=9, *N*=3) mice. **(C)** Representative traces showing the relaxation induced by 0.01–30μm retigabine in rings isolated from young, 12-, and 24-month-old mice. **(D)** Concentration-response relationships for retigabine-induced relaxation in mesenteric arteries in young (*n*=12, *N*=3), and 24-month-old (*n*=7, *N*=2) mice. ^*^p<0.05. Two-way ANOVA followed by Tukey *post hoc* test. Data are mean and SEM.

Next, we assessed the role of K_V_7 channels during aging. K_V_7 channels were activated by flupirtine and retigabine, which are considered as potent KCNQ3-5 activators in vascular smooth muscle (Tsvetkov et al., 2017). Flupirtine produced concentration-dependent relaxations; however, the effects were reduced by increased age. For instance, in arterial rings from 12- and 24-month-old mice, the effects were clearly age-dependent (Figures 2A,B). The 95% CI for EC_50_ of young, 12-, and 24-month-old mice rings were 0.6–0.8μm, 1.8–4.8μm, and 12.4–43.3μm, respectively. Retigabine caused similar effects (Figures 2C,D).

**Figure 3:**
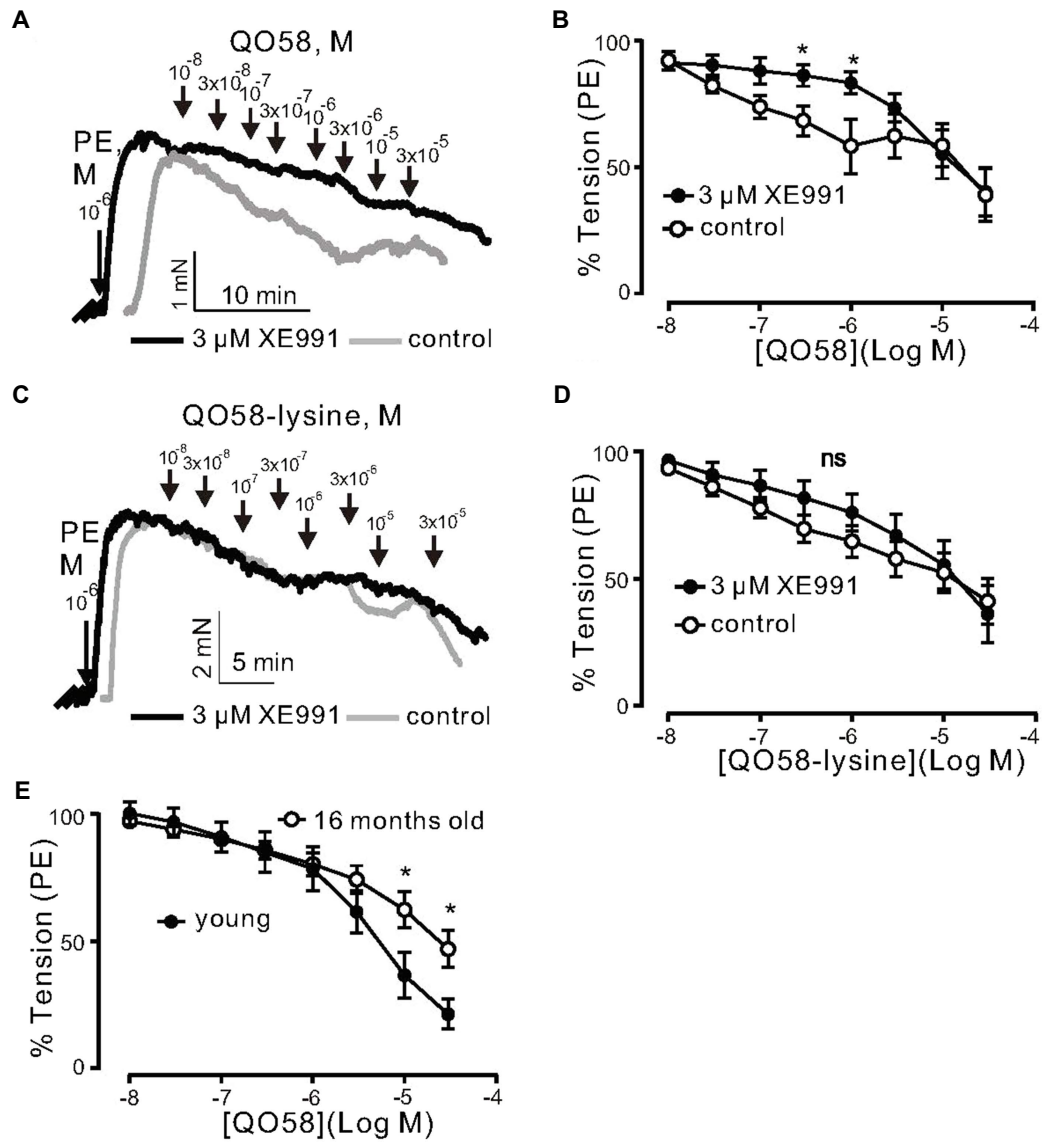
Relaxation of (−) PVAT mesenteric artery rings by novel KCNQ channel openers QO58 and QO58-lysine. **(A)** Original traces showing the effects of 3μM XE991 on QO58-induced relaxation in (−) PVAT mesenteric artery rings compared with control rings without XE991. **(B)** Concentration-response relationships for QO58-induced relaxation in (−) PVAT mesenteric arteries from young wild-type animals after pre-incubation with 3μm XE991 (*n*=10, *N*=4) or in the absence of XE991 (*n*=6, *N*=3). **(C)** Original traces showing the effects of 3μm XE991 on QO58-lysine-induced relaxation in (−) PVAT mesenteric artery rings compared with control rings without XE991. **(D)** Concentration-response relationships for QO58-lysine-induced relaxation in (−) PVAT mesenteric arteries from young animals after pre-incubation with 3μm XE991 (*n*=10, *N*=4) or in the absence of XE991 (*n*=9, *N*=3). **(E)** Concentration-response relationships for QO58-induced relaxation in mesenteric arteries in young (*n*=11, *N*=2) and 16-month-old (*n*=13, *N*=2) mice. ^*^p<0.05. Unpaired *t* test. Data are mean and SEM.

We tested two novel K_V_7 channel activators, namely, QO58 and QO58-lysine. QO58 produced concentration-dependent relaxations. The effects were abolished by 3μM XE991 (pan K_V_7 channel blocker) at low QO58 concentrations (<1μm; Figures 3A,B). In contrast, XE991 was unable to inhibit relaxations induced by QO58-lysine (Figures 3C,D). The data suggest that OQ58 but not QO58-lysine is capable of producing arterial relaxations through activation of XE991 sensitive KCNQ channels. Similar to flupirtine and retigabine, aging attenuated QO58-induced relaxations (Figure 3E). Aged mice mesenteric arteries displayed normal resting membrane potential (Supplementary Figures S1A–C). However, 3μm QO58 caused hyperpolarization of the membrane potential only in young mice; this hyperpolarization was reversed by 3μm XE991 in young (Supplementary Figures S1D,E) but not in old arteries (Supplementary Figures S1F,G). Simultaneously measured tension confirmed previously obtained results whereas XE991-induced depolarization led to contraction of young but not old vessels (Supplementary Figures S1D,F,H; Figures 3A,B). Thus, our data indicate that KCNQ channel function is impaired in aging.

**Figure 4:**
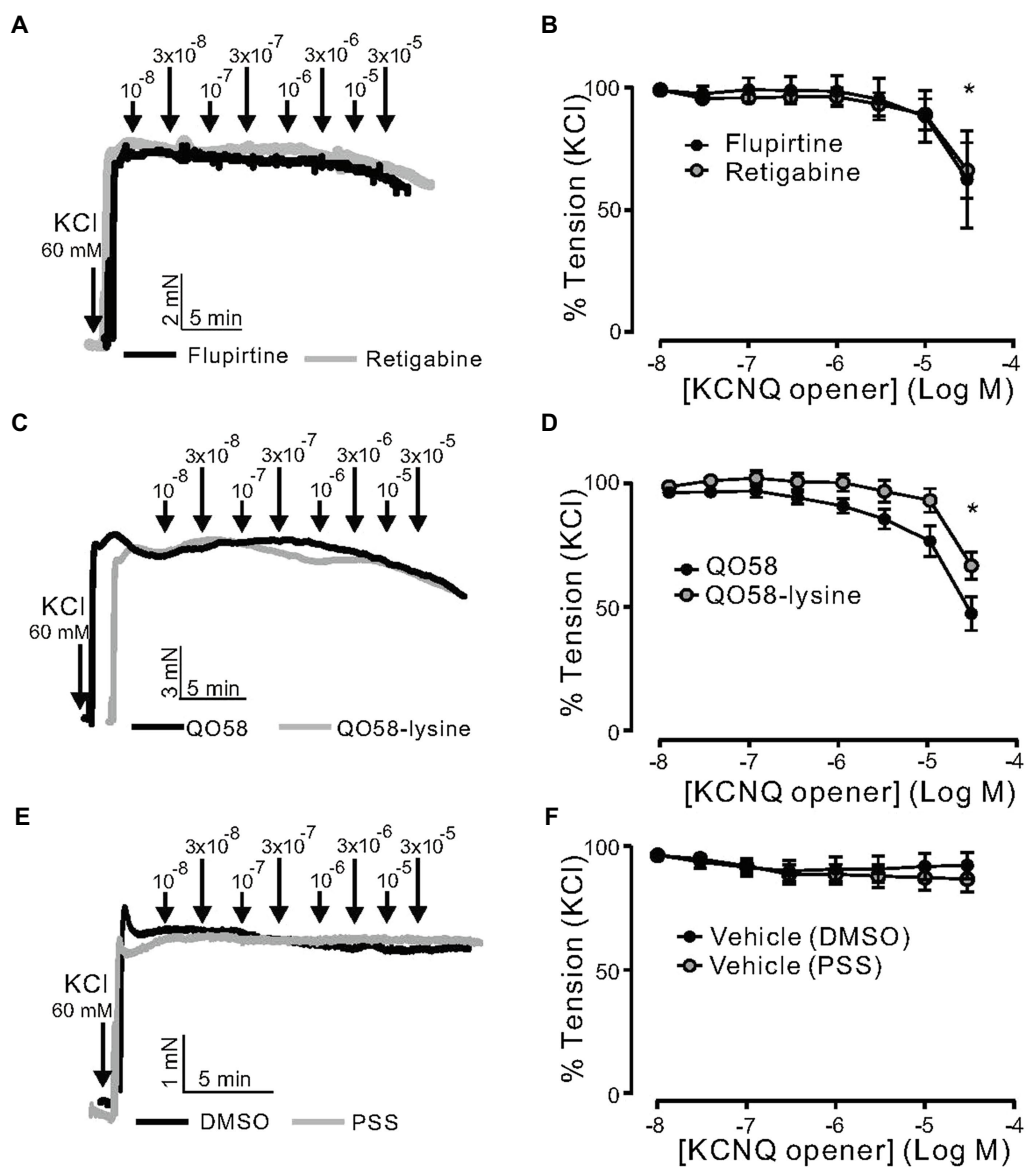
KCNQ channel openers effects on KCl-induced contraction in (−) PVAT mesenteric artery rings. **(A)** Original recordings showing the effects of 0.01–30μm retigabine, 0.01–30μm flupirtine on arterial tone of isolated mesenteric artery rings without (−) PVAT. Vessels were precontracted with 60mM KCl. **(B)** Concentration-response relationships for flupirtine- (*n*=6, *N*=2) and retigabine-induced relaxation (*n*=7, *N*=2) in (−) PVAT mesenteric arteries from young wild-type animals. **(C)** Original recordings showing the effects of 0.01–30μm QO58 and 0.01–30μm QO58-lysine on arterial tone of isolated mesenteric artery rings without (−) PVAT. **(D)** Concentration-response relationships for QO58 (*n*=9, *N*=3) and QO58-lysine-induced relaxation (*n*=8, *N*=4) in (−) PVAT mesenteric arteries from young wild-type animals. **(E)** Original recordings showing the effects of vehicle (DMSO or PSS) on arterial tone of isolated mesenteric artery rings without (−) PVAT. **(F)** Concentration-response relationships for DMSO (*n*=5, *N*=2) and PSS (*n*=5, *N*=2) in (−) PVAT mesenteric arteries from young wild-type animals. ^*^p<0.05. paired sample *t* test. Data are mean and SEM.

Then, we determined whether or not the effects of flupirtine, retigabine, QO58, and QO58-lysine rely on K^+^ channel activation. Raising external [K^+^] to 60mm would be expected to diminish the effects of any K^+^ channel opener by substantially reducing the difference between the potassium equilibrium potential and membrane potential. In these conditions, contractions are primarily caused by Ca^2+^ influx through L-type Ca_V_1.2 channels resistant to K^+^ channel openers (Essin et al., 2007). We found that flupirtine, retigabine, QO58, and QO58-lysine produced moderate relaxation only at relatively high (≥30μm) concentrations (Figures 4A–D). Vehicle application produced no relaxations (Figures 4E,F). Therefore, all four KCNQ channel activators may have off-target effects either on downstream targets regulating Ca^2+^ channels or L-type Ca_V_1.2 channels itself, only at higher concentrations (≥ 30μm).

### RNA Sequencing

**Figure 5:**
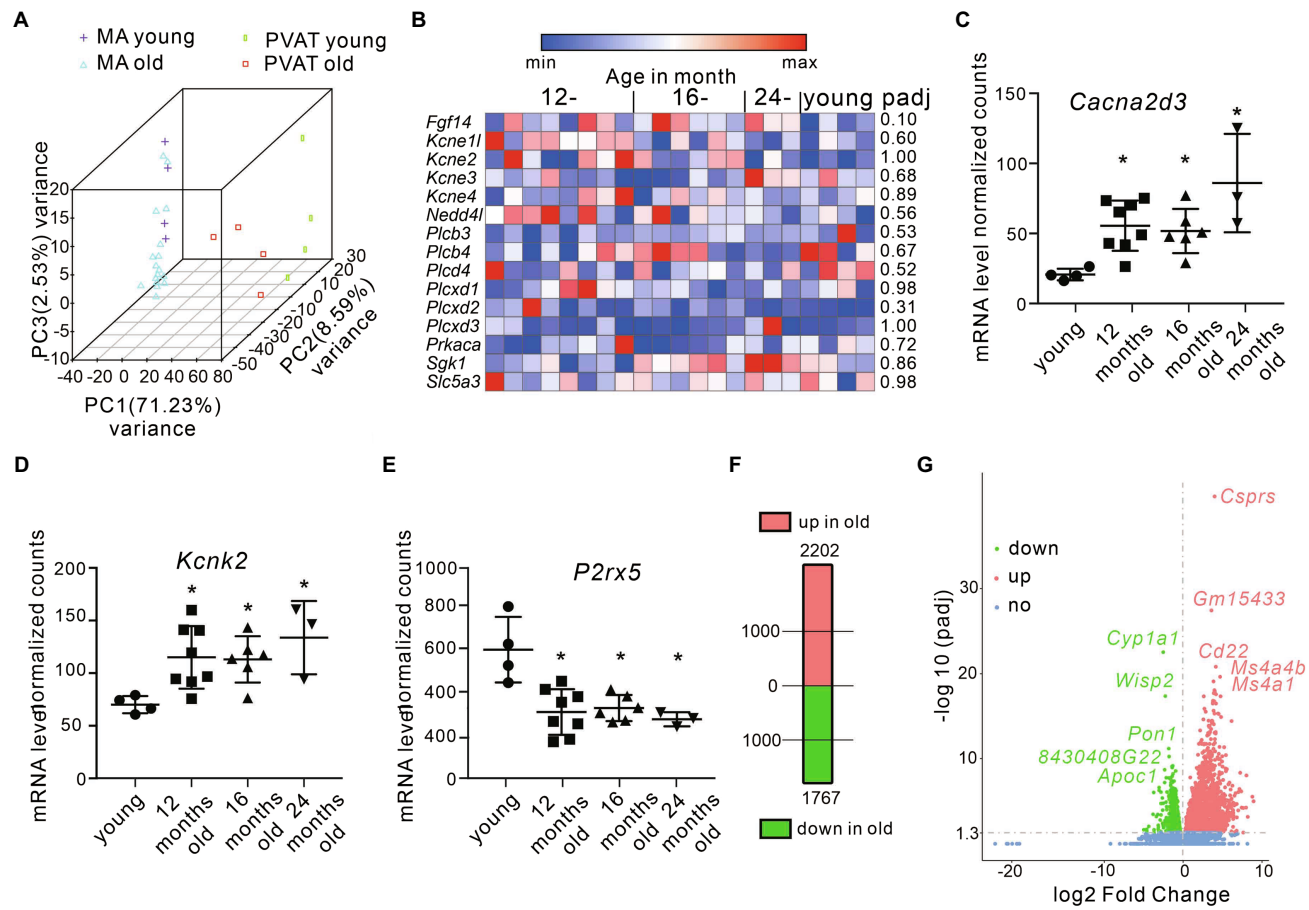
Differential gene expression in mesenteric arteries and PVAT from young and old mice. **(A)** Principal component analysis in samples isolated from young vs. old animals. **(B)** Heatmap showing the expression of candidate genes involved in pathways regulating KCNQ channels (*N*=8 for 12-month-old; *N*=6 for 16-month-old; *N*=3 for 24-month-old; *N*=4 for young mice). Data are fragments per kilobase per million mapped fragments. **(C)** Relative mRNA levels for *Cacna2d3* (*N*=4 for young; *N*=8 for 12-month-old; *N*=6 for 16-month-old; *N*=3 for 24-month-old mice). **(D)** Relative mRNA levels for *Kcnk2* (*N*=4 for young; *N*=8 for 12-month-old; *N*=6 for 16-month-old; *N*=3 for 24-month-old mice). **(E)** Relative mRNA levels for *P2rx5* (*N*=4 for young; *N*=8 for 12-month-old; *N*=6 for 16-month-old; *N*=3 for 24-month-old mice). **(F)** Number of differentially expressed genes in PVAT isolated from mesenteric arteries in young and old mice. **(G)** Volcano plot displaying statistical significance (adjusted value of *p*) versus magnitude of change (fold change) for differently expressed genes in PVAT isolated from 12-month-old (*N*=4) vs. young mice (*N*=4). Top 5 differentially expressed genes are marked. ^*^p<0.05. Wald test. Data are mean and SD. Abbreviations can be found in Supplementary Table S3.

To examine age-related changes in mRNA expression in mesenteric arteries and PVAT, we performed targeted and bulk RNA sequencing (RNA-seq) utilizing arterial tissue from young and old mice. Per sample, we obtained 23±2.5 million reads. ~97,5% of all reads were mapped to the reference mouse genome (ensembl_mus_musculus_grcm38_p6_gca_000001635_8). The principal component analysis (PCA) demonstrated tight clustering within each group and transcriptome difference between groups (Figure 5A). In (−) PVAT mesenteric arteries isolated from 12–16-, and 24-month-old mice, we were interested in candidate genes involved in pathways regulating KCNQ channels. Figure 5B shows the results. The data show that none of the genes were affected by aging. However, we found that transcripts of several ion channels were up- or downregulated in (−) PVAT mesenteric arteries during aging. The results are shown in Figures 5C–E. Of note, the mRNA expression of *Kcnq1,3,4,5* was normal across the different ages. We also confirmed these results using qPCR (Supplementary Figure S2). In PVAT from 12-month-old mice, 2,202 transcripts were upregulated and 1767 were downregulated (Figure 5F). Top 5 down- and upregulated genes are depicted on Figure 5G.

### Metabolic and Inflammatory Pathways

**Figure 6:**
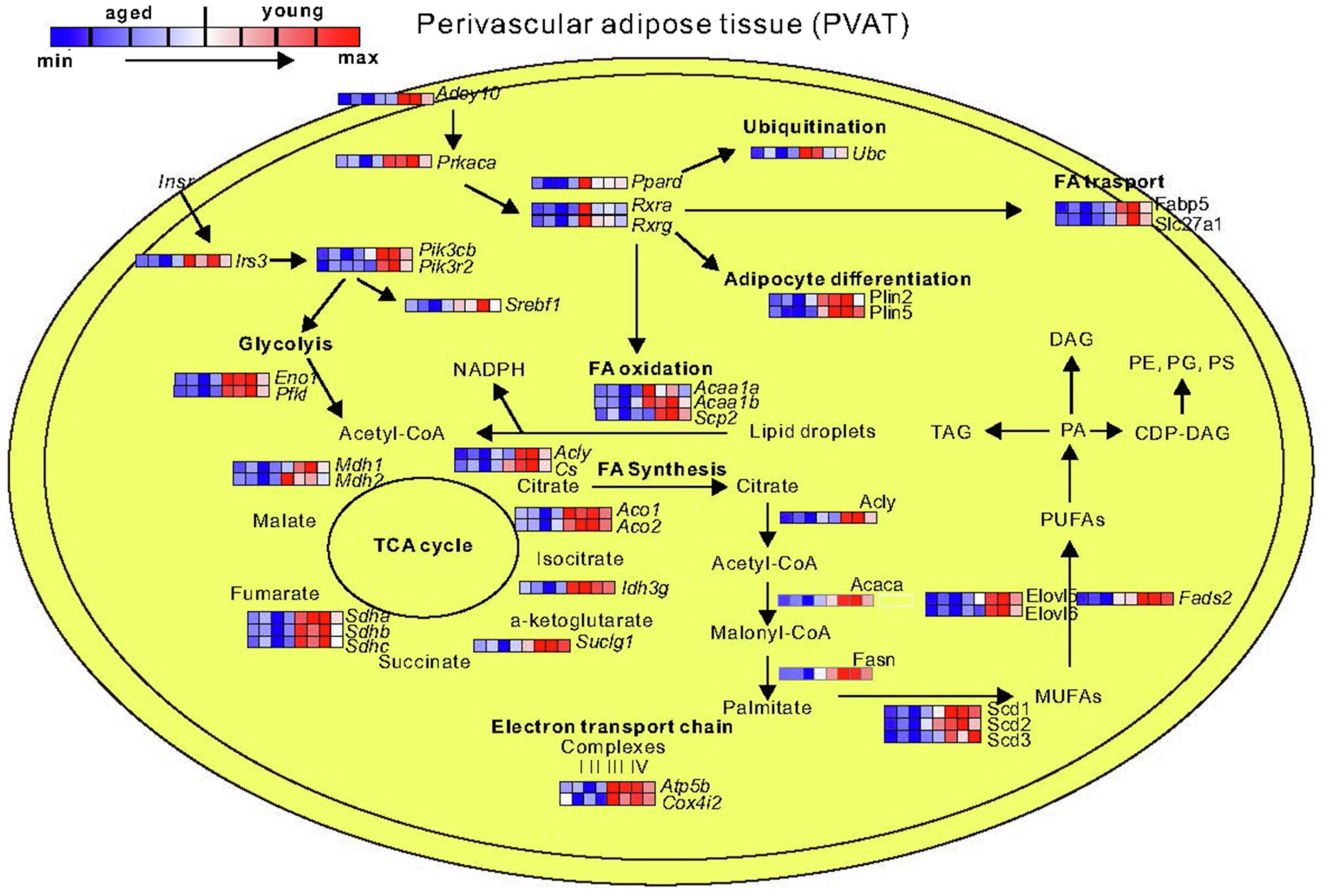
Representative schematic of downregulated genes and pathways in PVAT isolated from mesenteric arteries of 12-month-old mice. By decreased expression of genes belonging to Ppar and Rcr signaling pathway, the ubiquitination, fatty acid (FA) transport, adipocyte differentiation, and FAs oxidation are decreased. In addition, the downregulated glycolysis, TCA cycle, and fatty acid biosynthesis can decrease production of perivascular fat tissue-derived relaxation factors (*N*=4 for young; *N*=4 for 12-month-old). MUFAs, monounsaturated fatty acids; PUFAs, polyunsaturated fatty acids; PA, phosphatidic acid; TAG, triacylglycerol; DAG, diacylglycerol; PE, phosphatidylethanolamine; PG, phosphatidylglycerol; PS, phosphatidylserine. Abbreviations can be found in Supplementary Table S4.

Next, we performed Gene Ontology (GO) enrichment analysis using biological process (BP) terms and KEGG pathways. Our data show that aged PVAT exhibited upregulated pathways associated with inflammatory processes (e.g., GO:0002250, GO:0051249, and GO:0002764; mmu05150, mmu05152, and mmu04060; Supplementary Table S1). Downregulated were mostly BP and pathways related to generation of precursor metabolites and energy (e.g., GO:0006091, GO:0051186, GO:0006119, mmu00190, mmu01212, and mmu03320; Supplementary Table S2). In detail, the downregulated genes include mitochondrial genes associated with Parkinson (mmu05012) and Huntington (mmu05016), fatty acid metabolism (mmu01212), biosynthesis of unsaturated fatty acids (mmu01040), fatty acid elongation (mmu00062), insulin signaling (mmu04910), and PPAR pathway (mmu03320; Figure 6).

## Discussion

We present several novel findings. First, we showed that the anti-contractile effects of PVAT are impaired in mouse mesenteric arteries with increased age. Second, we observed altered functional role of K_V_7 (KCNQ) channels during aging. Finally, aging-related transcriptome changes in mesenteric arteries and PVAT uncovered possible downstream targets of PVAT signaling pathway. Altogether, our results provide novel insights into cardiovascular events associated with aging.

Alterations in PVAT contribute to vascular dysfunction in obesity, hypertension, and cardiometabolic disease in animal models and humans (Greenstein et al., 2009; Galvez-Prieto et al., 2012). We found that the anticontractile effects of PVAT are diminished in mouse mesenteric arteries with increased age. In addition, we observed morphological changes of mesenteric PVAT during aging. PVAT cells from old mice were increased in size (Supplementary Figure S3). In general, hypertrophic fat cells are considered less metabolically favorable and can produce inflammatory cytokines (Stenkula and Erlanson-Albertsson, 2018). Furthermore, Ly6B inflammatory cell infiltration was higher in old PVAT (Supplementary Figure S4). Previous study showed that also rat aortic PVAT composition, namely, decrease of browning, is associated with vascular dysfunction during aging in spontaneous hypertensive rats (Kong et al., 2018). We conclude from these data that adipose-vascular uncoupling undergoes age-dependent changes during life span. Agabiti-Rosei et al. (2017) also found that the anticontractile effects of PVAT are abolished in mesenteric arteries from aging SAMP8 mice, which is a senescence-accelerated prone mouse model (Agabiti-Rosei et al., 2017). These findings support the notion that restoring adipose-vascular coupling could be a promising therapeutic strategy in vascular aging. We further investigated K_V_7 family of K^+^ channels as putative downstream targets of relaxing factors released by PVAT (Schleifenbaum et al., 2010; Tsvetkov et al., 2017). Our data show that K_V_7.3–5 channel opening by flupirtine and retigabine induces relaxation in mesenteric arteries from young and old mice, making K_V_7.3–5 channels a possible therapeutic target for hypertension treatment in the elderly. Similar effects were observed for QO58, which is a novel KCNQ channel activator. In whole-cell patch-clamp and cell culture experiments, QO58 demonstrates high potency of opening KCNQ channels (for K_V_7.4 EC_50_=0.6μm, for K_V_7.3/7.5 EC_50_=5.2μm; Zhang et al., 2013). Thus, QO58 might be a promising tool for translational research in vascular biology. Although QO58-lysine modification resulted in an improved bioavailability of the drug (Teng et al., 2016), our results in whole artery preparations argue against specificity of QO58-lysine to be capable to open KCNQ channels in intact vascular tissue. Nevertheless, our data demonstrate that KCNQ channel induced relaxations by the three K_V_7 channel openers were more attenuated in tissue from aged mice. Moreover, electrophysiological data showed that QO58 causes hyperpolarization of the membrane potential, and this effect is reversed by XE991 in young mice. In contrast, pharmacological modulators of K_V_7 channels produced no changes of membrane potential in aged mice implying that K_V_7 function is altered in aging (Figure 2; Supplementary Figure S1). We found that *Kcnq1,3,4,5* mRNA expression was unchanged in the arteries during aging. These RNA-seq findings were confirmed by qPCR (Supplementary Figure S2). Thus, we concluded that impaired relaxation caused by KCNQ channels activation is not due to their changes in mRNA expression.

The RNA-Seq did not reveal additional targets or pathways. For instance, mRNA gene expressions already known to regulate KCNQ channel function (Supplementary Table S5) were similar in aged mice (Figure 5B). Thus, other mechanisms, such as post-translational modification (PTM) or trafficking, could be responsible for age-associated KCNQ channel dysfunction. Noteworthy, PTM is a new immerging paradigm of acquired channelopathies that can occur in congestive heart failure (Curran and Mohler, 2015). Post-translational modification of ion channels, such as voltage-dependent Na channels, is observed in chronic pain syndrome (Laedermann et al., 2015). Future studies are necessary to clarify PTM’s contribution to regulation of vascular tone in aging.

Nonetheless, RNA-Seq revealed activation of inflammatory process in old mice in mesenteric arteries. To our knowledge, this study is the first to firmly establish inflammatory transcriptome profile during different age using small resistant arteries (diameters: 150–200μm). Our data are also consistent with the idea that inflammation is one of the key mechanisms causing vascular damage in mouse-aged aorta (Gao et al., 2020). In addition, Th17-dependent immune response was activated (Table S1). In line with our previous findings, Th17 axis plays an important role in increased blood pressure (Wilck et al., 2017). Importantly, the anticontractile properties of PVAT can be restored in aging by melatonin treatment associated with decreased oxidative stress and inflammatory reaction (Agabiti-Rosei et al., 2017). Furthermore, anti-inflammatory therapy targeting the interleukin-1β innate immunity pathway in patients significantly decreased rate of recurrent cardiovascular events (Ridker et al., 2017). The participants were 60years old on average and number needed to treat was relatively large (~20). Since middle-aged human (38–47years) equivalents to 1-year-old mouse, one could argue that anti-inflammatory interventions in human should be started earlier, in order to achieve better outcome (Flurkey et al., 2007). Moreover, in the later phase of aging, remodeling takes place as shown by upregulated GO:0030198 and extracellular matrix organization (Supplementary Table S1). Similar results were found in mouse aorta (Gao et al., 2020), suggesting that vasculature damage caused by low-grade inflammation is a common process in aging.

Previous studies identified NAD^+^ precursor nicotinamide mononucleotide as activator of sirtuin deacylases and as a tool to reverse vascular aging (Das et al., 2018). By showing downregulation of pathway associated with energy production and therefore production of NAD and NAD precursors in mesenteric arteries of 16-month-old mice (Supplementary Table S2), our study contributes to the debate about the importance of NAD-dependent activity of sirtuin deacylases in aging. Interestingly, PVAT transcriptional profile in our study resembles visceral fat in insulin resistance patients. For example, downregulated mitochondrial respiratory and lipid metabolic pathways were found in obese insulin-resistant subjects (Soronen et al., 2012). We observed similar pattern in PVAT genes involved into fatty acid, cholesterol, and triglyceride metabolism (*Fatp2*, *Elovl6*, *Srebf1*). *Db/db* gene-deficient mice exhibited decreased expression of *Srebf1*, which was associated with impaired anticontractile effects of PVAT (Yahagi et al., 2002; Meijer et al., 2013). Similar results were obtained at the protein level using adipose tissue proteomic profiling in aged mice (Supplementary Table S6; Yu et al., 2020). The proper function of these metabolic pathways might be essential for producing PVATRFs. Although their nature is still a mystery, several proteins and lipids released by PVAT have vasodilatory properties. This state-of-affairs was previously reviewed in detail (Fernandez-Alfonso et al., 2017). Such palmitic acid methyl ester (PAME) has been proposed as transferable PVATRF in rat aorta (Lee et al., 2011). However, PAME could contribute to PVAT-induced relaxations by activating K_V_7 channels in rat aorta, but not in human mesenteric arteries (Wang et al., 2018). Interestingly, omega 3 epoxide of docosahexaenoic acid (DHA) can open two-pore domain K+ channels and lower blood pressure (Nielsen et al., 2013; Ulu et al., 2014). However, whether it is indeed a PVATRF remains to be clarified. Similar metabolic pathways may control smooth muscle cell differentiation through subset of PVAT-derived stem cells (Gu et al., 2019). Thus, in addition to existing criteria such as Ca^2+^ dependence for PVATRFs, these factors could represent metabolites of fatty acids biosynthesis. Consequently, PVATRFs concentration should decrease during aging. Furthermore, PPAR pathway is compromised through peroxisome proliferator-activated receptor-γ coactivator-1 α (Ppargc1a) and lead to vascular remodeling during aging *via* decreased brown adipogenic differentiation in PVAT isolated from aorta (Pan et al., 2019). Our data indicate that PPAR pathway is also downregulated in PVAT surrounding mouse mesenteric arteries. However, the mechanism does unlikely involve *Ppargc1a* mRNA, since its expression was similar in aged and young mice (fold change=0.27, padj=0.47). A functionally distinct vessel type could explain this difference.

We studied mRNA transcript differences of several ion channels in aging vessels. Only three transcripts, namely, upregulated *Cacna2d3*, *Kcnk2* and downregulated *P2rx5*, intersected all three data sets (12-, 16-, and 24-month-old mice). We speculate that these ion channels could represent novel putative targets of arterial tone regulation. For example, auxiliary voltage-dependent calcium channel subunits delta (Cacna2d) contribute to trafficking and proper surface expression of voltage-gated calcium channels (VGCCs, Ca_V_2; Dolphin, 2012). These channels are responsible for the P/Q current in and therefore could be of great importance for blood pressure regulation (Andreasen et al., 2006). Interestingly, *Cacna2d3 knock out* mice exhibit reduced L-type and N-type currents in spiral ganglion neurons (Stephani et al., 2019). Thus, although the vascular phenotype of *Cacna2d3*-deficient mice is not yet characterized, *Cacna2d3* arises as a novel candidate for increased blood pressure during aging. *Kcnk2* is known as TREK-1 (tandem of P domains in a weak inward-rectifier-related K^+^) channel. The channel has been implicated to play an important role in the brain vasculature (Blondeau et al., 2007). TREK-1 opening characteristics (e.g., activation by PUFAs) elevates the family as possible new targets for PVATRFs. Of note, TREK-1-deficient mice display endothelial dysfunction with decreased relaxation of mesenteric arteries (Garry et al., 2007). However, the anticontractile effect of PVAT has remained to be studied in these mice. *P2rx5* is a purinoceptor for ATP acting as ligand-gated ion channel. Vascular smooth muscle cells from mesenteric arteries express the P2X receptors. Though no evidence was found for a phenotype corresponding to homomeric P2X5 receptors or to heteromeric P2X1/5 receptors, the functional role of these receptors in arteries is still unclear (Lewis and Evans, 2000). Under inflammatory conditions, osteoclasts of P2rx5 gene-deficient mice have deficits in inflammasome activation and osteoclast maturation (Kim et al., 2017). However, their vascular phenotype has not yet been studied.

## Data Availability Statement

The data presented in the study are deposited in the figshare repository, accession number 16920589 https://figshare.com/articles/dataset/raw_data_of_Aging_Affects_KV7_Channels_and_Perivascular_Adipose_Tissue-Mediated_Vascular_Tone/16920589.

## Ethics Statement

The animal study was reviewed and approved by Landesamt für Gesundheit 69 und Soziales Berlin, LAGeSo. Animal care was followed by American Physiological Society guidelines, and local authorities.

## Author Contributions

YW, FY, AS, MK, LM, MK, FCL, MG, and DT were responsible for data collection, analysis, and interpretation. YW and DT drafted the manuscript. All authors have approved the final version of the manuscript and agreed to be accountable for all aspects of the work. All persons designated as authors qualify for authorship, and all those who qualify for authorship are listed.

## Funding

This work was supported by Deutsche Forschungsgemeinschaft (DFG, grant no 193179237); Deutsche Akademische Austauschdienst (DAAD); Chinese Scholarship Council.

## Conflict of Interest

The authors declare that the research was conducted in the absence of any commercial or financial relationships that could be construed as a potential conflict of interest.

## Publisher’s Note

All claims expressed in this article are solely those of the authors and do not necessarily represent those of their affiliated organizations, or those of the publisher, the editors and the reviewers. Any product that may be evaluated in this article, or claim that may be made by its manufacturer, is not guaranteed or endorsed by the publisher.

## Footnotes

^1^ https://software.broadinstitute.org/morpheus
