# Supplementary material for: Aging Affects KV7 Channels and Perivascular Adipose Tissue-Mediated Vascular Tone
Source: Front Physiol. 2021 Nov 26;12:749709. doi: 10.3389/fphys.2021.749709 (PMC8662361; doi:10.3389/fphys.2021.749709)
Supplement: Supplementary file 1 [file Data_Sheet_1.pdf]

SUPPLEMENTARY MATERIAL
Aging Affects K_V7 Channels and Perivascular Adipose Tissue-Mediated Vascular Tone

Figure S1.

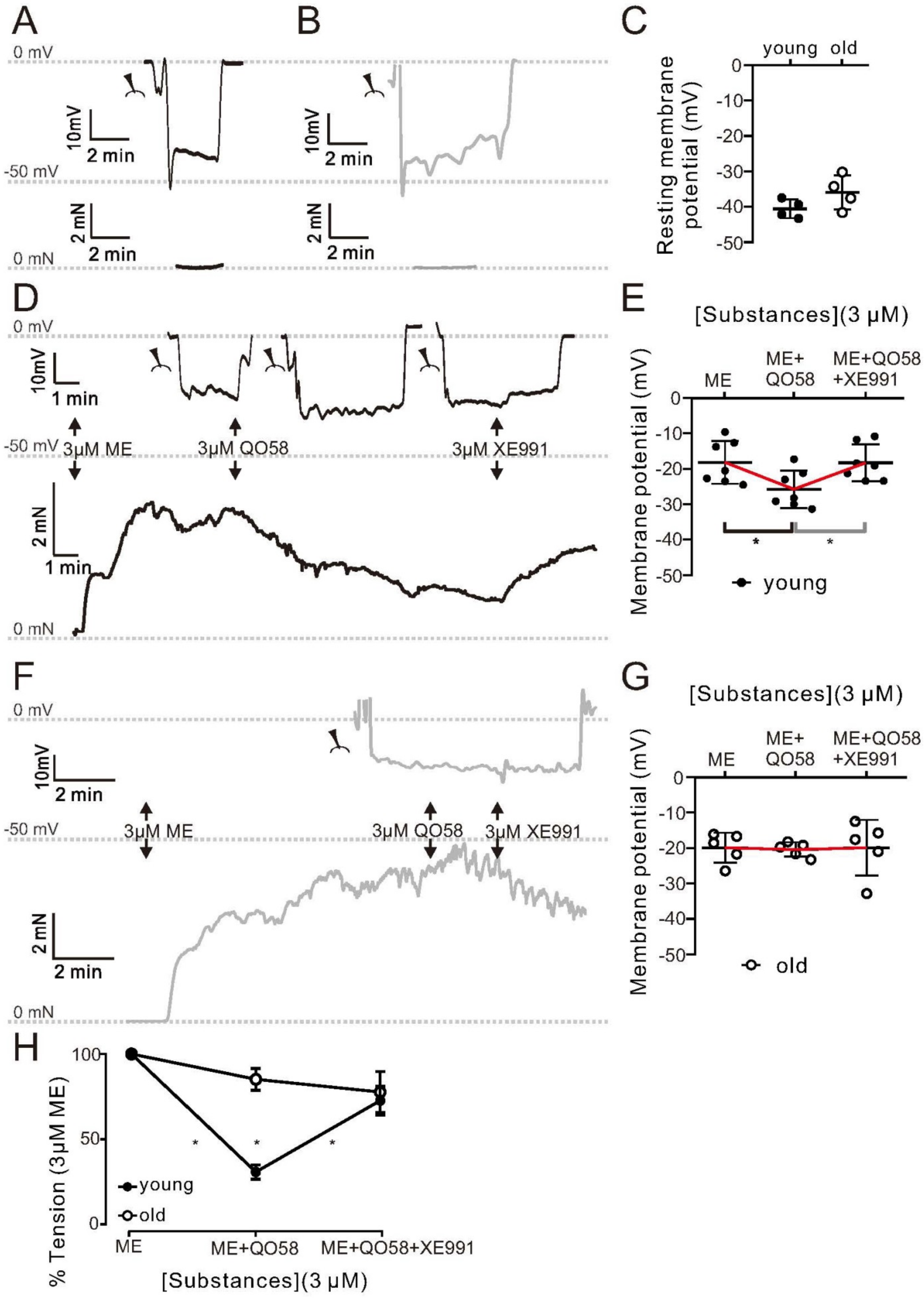

Figure S1. Aging-effects K_V7 regulation of membrane potential in mesenteric arteries

Example of resting membrane potential (upper trace) and contractile force (lower trace) in young (A) and old (B) mice. (C) Summarized data of resting membrane potential. Membrane potential (upper trace) and contractile force (lower trace) in isometric vessel preparation at 3µM methoxamine (ME)–induced tone and after subsequent application of 3 µM QO58 and 3 µM XE991 in young (D, E) and old (F, G) mice. The microelectrode symbol denotes phases when the microelectrode was impaled. Summarized data of contractile force (H) in the presence of 3µM ME, 3 µM ME + 3 µM QO58, and ME + QO58 + 3 µM XE991. (Data are mean and SD. (C): unpaired *t*-test n, N=4, for young and old mice; (E,G) *p<0.05. One-way ANOVA test with post-hoc Dunn's multiple comparison test; n=7, N=7 for young, n=5, N=5 for old mice, (H) *p<0.05. young ME vs. young ME + QO58; young ME + QO58 vs young ME+ QO58 + XE991; young ME + QO58 vs. old ME + QO58. Two-way ANOVA test with post-hoc Sidak multiple comparison test. n=7, N=7 for young, n=5, N=5 for old mice)

Figure S2.

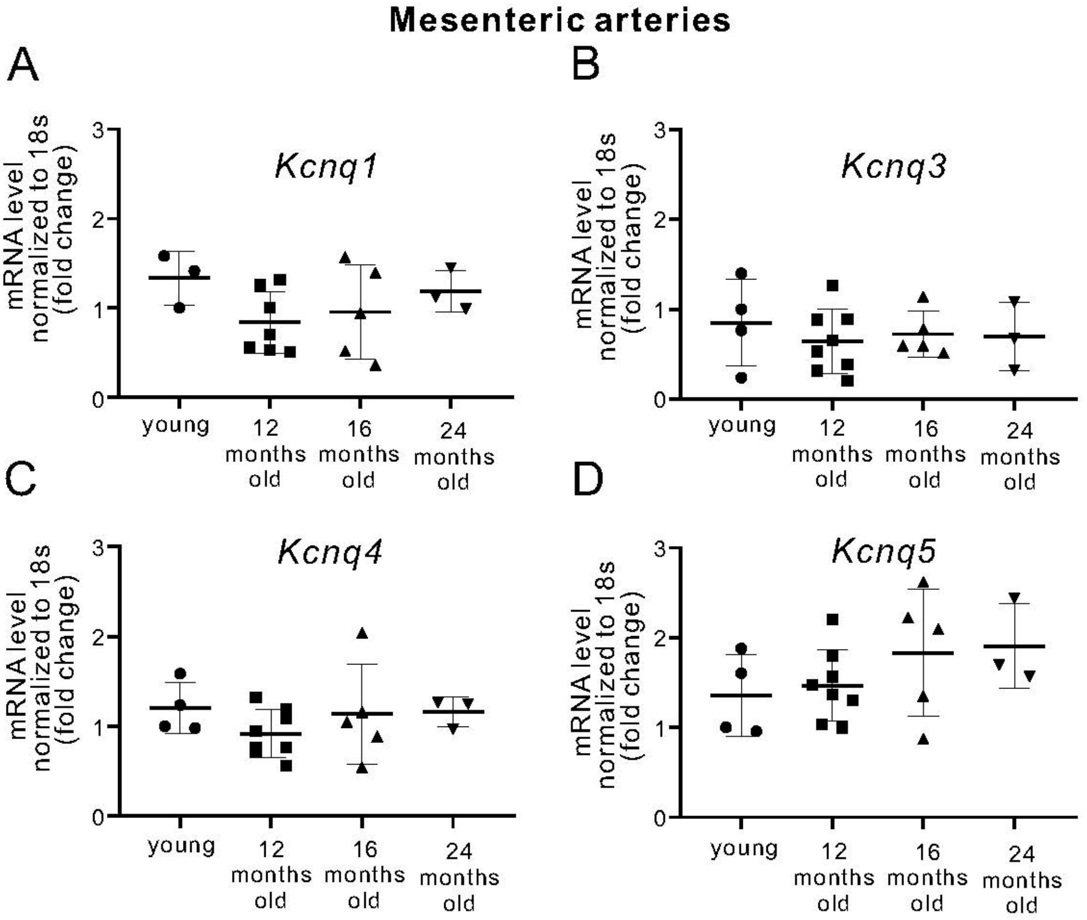

Figure S2. Relative expression of KCNQ 1, 3, 4, 5 channels at mRNA levels in (–) PVAT mesenteric arteries from young and aged mice normalized to 18s.

(A) Relative mRNA levels for Kcnq1 (N = 3 for young; N = 7 for 12-months old; N = 5 for 16-months old; N = 3 for 24 months old mice).

(B) Relative mRNA levels for Kcnq3 (N = 4 for young; N = 8 for 12-months old; N = 5 for 16-months old; N = 3 for 24-months old mice).

(C) Relative mRNA levels for Kcnq4 (N = 4 for young; N = 8 for 12-months old; N = 5, for 16-months old; N = 3 for 24-months old mice).

(D) Relative mRNA levels for Kcnq5 (N = 4 for young; N = 8 for 12-months old; N = 5 for 16-months old; N = 3 for 24-months old mice). ns, $P > 0.05$, Kruskal–Wallis one-way analysis of variance. Data are mean and SD.

Figure S3.

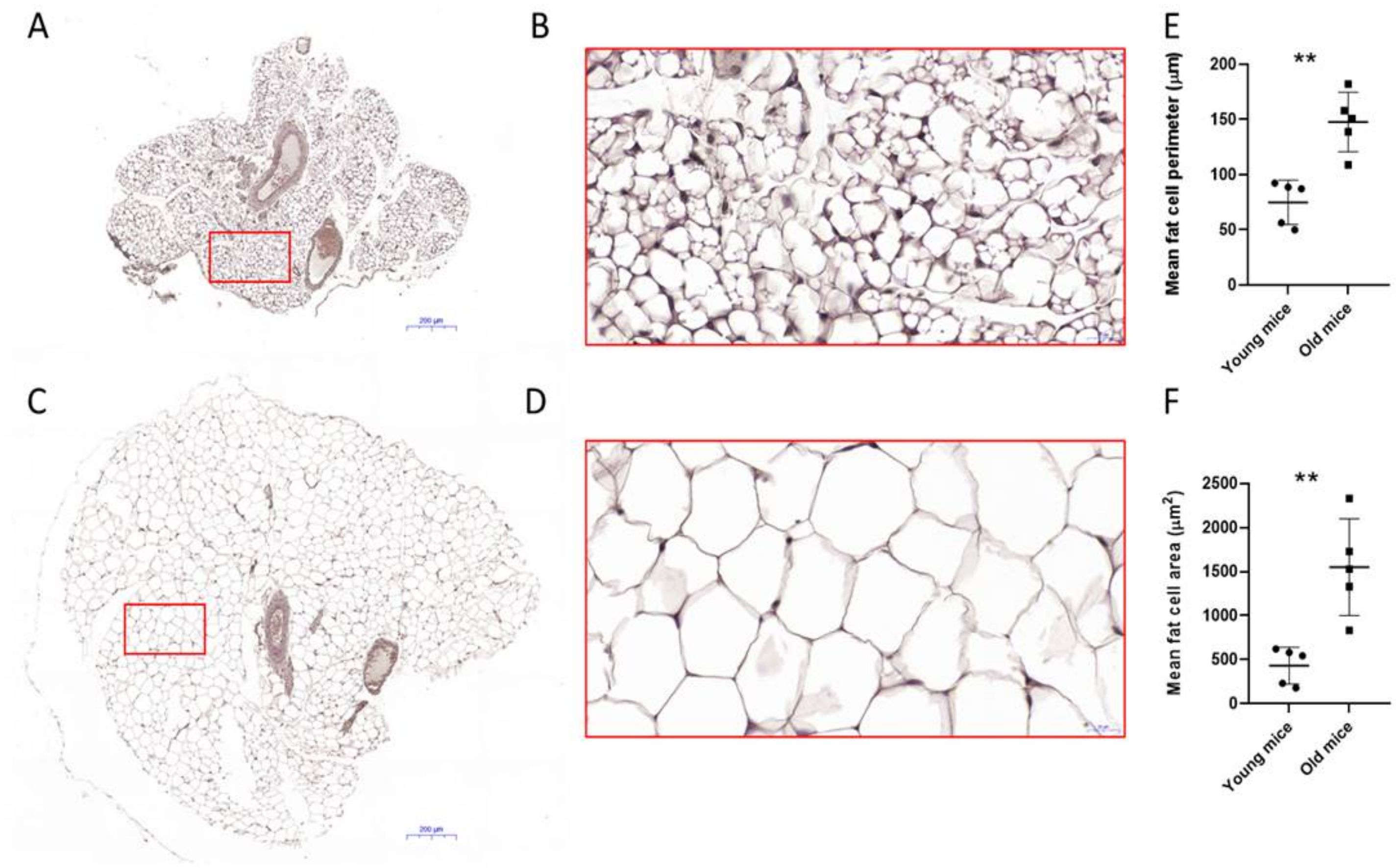

Figure S3. Age-associated changes in PVAT. Hematoxylin and eosin stain of PVAT around mesenterial arteries and veins of young (A and B) and old (C and D) mice. Mean fat cell per perimeter (C) and area (F). Magnification 5x, scale bar=200µm (A and C) and 40x, scale bar = 20µm (B and D). Data are mean and SD. *p<0.01, two-sided unpaired *t*-test.

Figure S4.

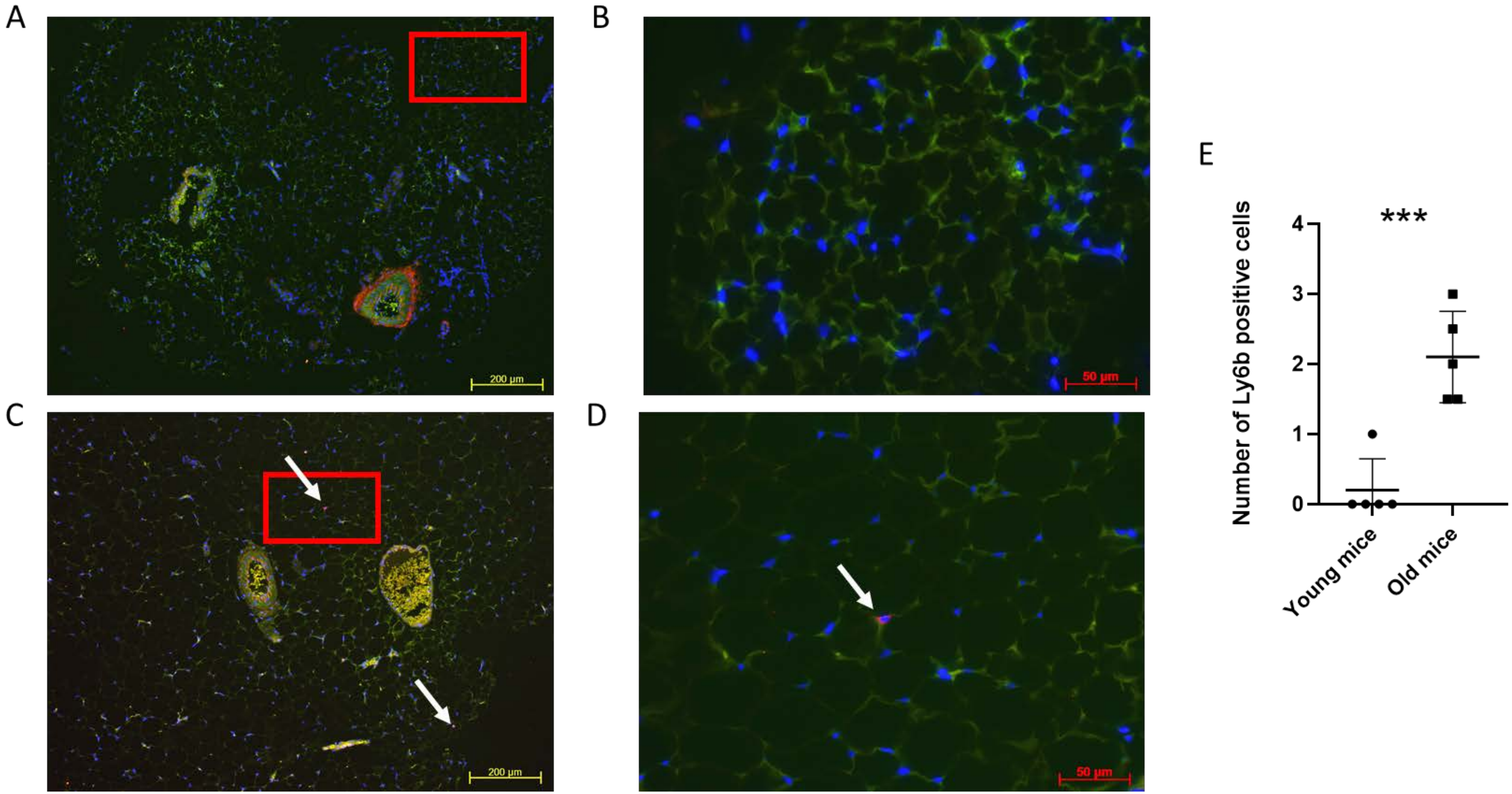

Figure S4. Ly6B stain (red) of perivascular fat around mesenterial vessels of young (A and B) and old (C and D) mice. Blue DAPI, green autofluoresence. Mean number of Ly6b positive cells of two sections (E). Magnification 10x, scale bar = 200µm (A and C) and magnification 40x, scale bar= 50 µm (B and D), ***p<0.001, two-sided unpaired t-test.

Table S1. Top upregulated GO Terms and KEGG Pathways in PVAT isolated from 12-month old mice

GO Biological Process			
**id**	**Terms**	**p. value**	**Adj.p.value**
GO:0002250	adaptive immune response	1.18E-90	6.11E-87
GO:0051249	regulation of lymphocyte activation	1.52E-75	3.91E-72
GO:0002764	immune response-regulating signaling pathway	5.80E-75	9.98E-72
GO:0002757	immune response-activating signal transduction	4.56E-72	5.89E-69
GO:0050867	positive regulation of cell activation	7.06E-71	7.30E-68
GO:0098542	defense response to other organism	9.32E-71	8.02E-68
GO:0002253	activation of immune response	2.83E-70	2.09E-67
GO:0002696	positive regulation of leukocyte activation	4.29E-69	2.77E-66
GO:0042113	B cell activation	1.70E-68	9.78E-66
GO:0002768	immune response-regulating cell surface receptor signaling pathway	6.12E-67	3.16E-64
GO:0051251	positive regulation of lymphocyte activation	1.54E-66	7.21E-64
GO:0002429	immune response-activating cell surface receptor signaling pathway	1.36E-64	5.85E-62
GO:0050851	antigen receptor-mediated signaling pathway	5.76E-64	2.29E-61
GO:0002460	adaptive immune response based on somatic recombination of immune receptors built from immunoglobulin superfamily domains	1.23E-63	4.53E-61
GO:0002443	leukocyte mediated immunity	2.14E-60	7.36E-58
GO:0002449	lymphocyte mediated immunity	1.72E-58	5.56E-56
GO:0050864	regulation of B cell activation	9.82E-57	2.98E-54
GO:0050853	B cell receptor signaling pathway	6.90E-56	1.98E-53
GO:0042110	T cell activation	1.30E-51	3.53E-49
GO:0002250	adaptive immune response	1.18E-90	6.11E-87
**KEGG**			
**id**	**Terms**	**p. value**	**Adj.p.value**
mmu04060	Cytokine-cytokine receptor interaction	1.35E-24	4.06E-22
mmu05340	Primary immunodeficiency	7.28E-23	1.09E-20
mmu04640	Hematopoietic cell lineage	3.45E-22	3.45E-20
mmu04672	Intestinal immune network for IgA production	2.17E-17	1.62E-15
mmu04380	Osteoclast differentiation	1.00E-16	6.02E-15
mmu04658	Th1 and Th2 cell differentiation	1.48E-16	7.42E-15
mmu05321	Inflammatory bowel disease (IBD)	2.26E-16	9.47E-15
mmu04064	NF-kappa B signaling pathway	2.52E-16	9.47E-15
mmu04650	Natural killer cell mediated cytotoxicity	3.40E-16	1.13E-14
mmu05330	Allograft rejection	1.17E-15	3.51E-14
mmu04660	T cell receptor signaling pathway	1.47E-15	4.02E-14
mmu04514	Cell adhesion molecules (CAMs)	1.82E-15	4.56E-14
mmu04659	Th17 cell differentiation	2.98E-15	6.89E-14
mmu04662	B cell receptor signaling pathway	3.09E-14	6.62E-13
mmu05332	Graft-versus-host disease	4.92E-14	9.85E-13
mmu04061	Viral protein interaction with cytokine and cytokine receptor	6.91E-14	1.30E-12
mmu05168	Herpes simplex virus 1 infection	6.48E-13	1.14E-11
mmu04940	Type I diabetes mellitus	2.12E-12	3.53E-11

mmu05140	Leishmaniasis	3.59E-12	5.67E-11
mmu04060	Cytokine-cytokine receptor interaction	1.35E-24	4.06E-22

Table S2. Top downregulated GO Terms and KEGG Pathways in PVAT isolated from 12-month old mice

GO Biological Process			
**id**	**Terms**	**p. value**	**Adj.p.value**
GO:0006091	generation of precursor metabolites and energy	7.32E-47	3.48E-43
GO:0051186	cofactor metabolic process	4.73E-41	1.12E-37
GO:0022900	electron transport chain	2.51E-39	3.98E-36
GO:0045333	cellular respiration	9.33E-39	1.11E-35
GO:0046034	ATP metabolic process	1.84E-36	1.75E-33
GO:0032787	monocarboxylic acid metabolic process	7.77E-36	6.15E-33
GO:0015980	energy derivation by oxidation of organic compounds	9.13E-35	6.20E-32
GO:0009161	ribonucleoside monophosphate metabolic process	4.17E-34	2.48E-31
GO:0009167	purine ribonucleoside monophosphate metabolic process	6.48E-34	3.42E-31
GO:0006119	oxidative phosphorylation	7.45E-34	3.54E-31
GO:0009199	ribonucleoside triphosphate metabolic process	8.44E-34	3.65E-31
GO:0009126	purine nucleoside monophosphate metabolic process	9.63E-34	3.81E-31
GO:0009205	purine ribonucleoside triphosphate metabolic process	2.11E-33	7.73E-31
GO:0009144	purine nucleoside triphosphate metabolic process	3.64E-33	1.23E-30
GO:0009123	nucleoside monophosphate metabolic process	6.08E-33	1.93E-30
GO:0022904	respiratory electron transport chain	1.38E-32	4.11E-30
GO:0009141	nucleoside triphosphate metabolic process	4.63E-32	1.29E-29
GO:0007005	mitochondrion organization	4.26E-31	1.12E-28
GO:0042773	ATP synthesis coupled electron transport	1.32E-30	3.30E-28
GO:0042775	mitochondrial ATP synthesis coupled electron transport	2.14E-30	5.08E-28
**KEGG**			
**id**	**Terms**	**p. value**	**Adj.p.value**
mmu05012	Parkinson disease	1.23E-41	3.79E-39
mmu00190	Oxidative phosphorylation	7.22E-41	1.11E-38
mmu05016	Huntington disease	3.95E-33	3.64E-31
mmu04932	Non-alcoholic fatty liver disease (NAFLD)	4.74E-33	3.64E-31
mmu04714	Thermogenesis	1.07E-30	6.59E-29
mmu01200	Carbon metabolism	9.08E-19	4.64E-17
mmu01212	Fatty acid metabolism	3.19E-18	1.40E-16
mmu04723	Retrograde endocannabinoid signaling	8.22E-15	3.16E-13
mmu04146	Peroxisome	2.66E-11	9.07E-10
mmu00620	Pyruvate metabolism	3.06E-11	9.40E-10
mmu03320	PPAR signaling pathway	3.68E-11	1.03E-09
mmu00020	Citrate cycle (TCA cycle)	2.73E-10	6.99E-09
mmu00280	Valine, leucine and isoleucine degradation	4.99E-10	1.18E-08
mmu00010	Glycolysis / Gluconeogenesis	2.95E-09	6.34E-08
mmu04260	Cardiac muscle contraction	3.10E-09	6.34E-08
mmu01040	Biosynthesis of unsaturated fatty acids	7.96E-09	1.53E-07

mmu01230	Biosynthesis of amino acids	4.98E-08	8.99E-07
mmu00062	Fatty acid elongation	6.78E-08	1.16E-06
mmu00640	Propanoate metabolism	3.46E-07	5.58E-06
mmu00900	Terpenoid backbone biosynthesis	5.77E-07	8.86E-06

Table S3. Abbreviations used in Figure 5

Gene Symbol	Gene Description
Fgf14	Fibroblast growth factor 14
Kcne1l	Potassium voltage-gated channel subfamily E regulatory beta subunit 5
Kcne2	Potassium voltage-gated channel subfamily E Isk-related subfamily 2
Kcne3	Potassium voltage-gated channel subfamily E member 3
Kcne4	Potassium voltage-gated channel subfamily E member 4
Nedd4l	Neural precursor cell expressed, developmentally down-regulated 4-like, E3 ubiquitin protein ligase
Plcb3	phospholipase C, beta 3
Plcb4	phospholipase C, beta 4
Plcd4	phospholipase C, delta 4
Plcxd1	PI-PLC X domain-containing protein 1
Plcxd2	PI-PLC X domain-containing protein 2
Plcxd3	Phosphatidylinositol-specific phospholipase C, X domain containing 3
Prkaca	protein kinase, cAMP dependent, catalytic, alpha
Sgk1	Serine/threonine-protein kinase Sgk1
Slc5a3	Sodium/myo-inositol cotransporter
Cyp1a1	Cytochrome P450 1A1
Wisp2	WNT1 inducible signaling pathway protein 2
Pon1	Paraoxonase 1
8430408G22	Protein DEPP1
Apoc1	Apolipoprotein C-I Truncated apolipoprotein C-I
Csprs	Component of Sp100-rs
Gm15433	predicted pseudogene 15433
Cd22	CD22 molecule
Ms4ab	Membrane-spanning 4-domains, subfamily A, member 4B
Ms4a1	Membrane-spanning 4-domains, subfamily A, member 1

Table S4. Abbreviations used in Figure 6

Gene Symbol	Gene Description
Adcy10	Adenylate cyclase type 10
Prkaca	Protein kinase, cAMP dependent, catalytic, alpha
Ppard	Peroxisome proliferator-activated receptor delta
Rxra	Retinoid X receptor alpha
Rxrg	Retinoic acid receptor RXR-gamma
Ubc	Ubiquitin C
Fabp5	Fatty acid-binding protein
Plin2	Perilipin-2

Plin5	Perilipin-5
Acaa1a	Acetyl-Coenzyme A acyltransferase 1A
Acaa1b	Acetyl-Coenzyme A acyltransferase 1B
Scp2	Sterol carrier protein 2
Insr	Insulin receptor
Irs3	Insulin receptor substrate 3
Pik3cb	Phosphatidylinositol 4,5-bisphosphate 3-kinase catalytic subunit beta isoform
Pik3r2	Phosphatidylinositol 3-kinase regulatory subunit beta
Srebf1	Sterol regulatory element-binding protein 1
Eno1	Alpha-enolase
Pfkl	ATP-dependent 6-phosphofructokinase
Acly	ATP citrate lyase
Cs	Citrate synthase
Aco1	Aconitase 1
Aco2	Aconitase 2
Idh3g	Isocitrate dehydrogenase 3 (NAD+), gamma
Suclg1	Succinate-CoA ligase [ADP/GDP-forming] subunit alpha
Sdha	Succinate dehydrogenase complex, subunit A
Sdhb	Succinate dehydrogenase complex, subunit B
Sdhc	Succinate dehydrogenase complex, subunit C
Mdh1	Malate dehydrogenase 1
Mdh2	Malate dehydrogenase 2
Acaca	Acetyl-Coenzyme A carboxylase alpha
Fasn	Fatty acid synthase
Atp5b	ATP synthase subunit beta
Cox4i2	Cytochrome c oxidase subunit 4 isoform 2
Scd1	Acyl-CoA desaturase 1
Scd2	Acyl-CoA desaturase 2
Scd3	Acyl-CoA desaturase 3
Fads2	Fatty acid desaturase 2
Elovl5	Elongation of very long-chain fatty acid protein 5
Elovl6	Elongation of very long-chain fatty acid protein 6

Table S5. Candidates involved in pathways regulating KCNQ channels

Candidate	Effect	Reference
FGF14	Positively regulates KCNQ channels	(1)
Kcne4	Alters Vascular Reactivity through modulating KCNQ channels	(2)
PIP_2	Regulates KCNQ channel openings	(Zaydman et al., 2013
cAMP/PKA	Enhance KCNQ currents	(3)
SGK-1 and Nedd4-2	Modulates KCNQ channels by SGK-1 regulation of the activity of the ubiquitin ligase Nedd4-2	(4)
SMIT1 or Slc5a3	Regulates KCNQ channel	(5, 6)

	ion selectivity	

Table S6. Significantly dysregulated genes in PVAT (RNA-Seq) and in white adipose tissue (WAT) (proteomics) in aging.

Gene Symbol (mRNA, from current study)	Gene Symbol (proteomics from (7))	Gene description	Expression	Process
Abhd14b	Abhd14b	Abhydrolase domain containing 14b	↓	Lipid Metabolism
Abhd6	Abhd6	Abhydrolase domain containing 6	↓	
Acaca	Acaca	Acetyl-Coenzyme A carboxylase alpha	↓	
Acacb	Acacb	Acetyl-Coenzyme A carboxylase beta	↓	
Echs1	Echs1	Enoyl-CoA hydratase, mitochondrial	↓	
Fasn	Fasn	Fatty acid synthase	↓	
Gpd2	Gpd2	Pleckstrin homology domain-containing family O member 1	↓	
Acly	Acly	ATP citrate lyase	↓	Central Carbon
Gls	Gls	Glutaminase kidney isoform, mitochondrial	↑	
Hk2	Hk2	Hexokinase-2	↓	
Mcee	Mcee	Methylmalonyl-CoA epimerase, mitochondrial	↓	
Pdhb	Pdhb	Pyruvate dehydrogenase E1 component subunit beta, mitochondrial	↓	
Pgk1	Pgk1	Phosphoglycerate kinase 1	↓	
Gpt2	Gpt2	Glutamic pyruvate transaminase	↓	
Hk3	Hk3	Hexokinase-3	↑	
Cox5b		Cytochrome c oxidase subunit 5B, mitochondrial	↓	
Cox6b1	Cox6b1	Cytochrome c oxidase subunit 6B1	↓	
Cox6c	Cox6c	Cytochrome c oxidase subunit 6C	↓	
Ndufa3	Ndufa3	NADH dehydrogenase [ubiquinone] 1 alpha subcomplex subunit 3	↓	
Ndufa4	Ndufa4	NADH dehydrogenase [ubiquinone] 1 alpha	↓	

		subcomplex subunit 4		Electron Transport Chain
Ndufa5	Ndufa5	NADH dehydrogenase [ubiquinone] 1 alpha subcomplex subunit 5	↓	
Ndufa6	Ndufa6	NADH dehydrogenase [ubiquinone] 1 alpha subcomplex subunit 6	↓	
Ndufa7	Ndufa7	NADH dehydrogenase [ubiquinone] 1 alpha subcomplex subunit 7	↓	
Ndufa8	Ndufa8	NADH dehydrogenase [ubiquinone] 1 alpha subcomplex subunit 8	↓	
Ndufa10	Ndufa10	NADH dehydrogenase [ubiquinone] 1 alpha subcomplex subunit 10	↓	
Ndufa11	Ndufa11	NADH dehydrogenase [ubiquinone] 1 alpha subcomplex subunit 11	↓	
Ndufa12	Ndufa12	NADH dehydrogenase [ubiquinone] 1 alpha subcomplex subunit 12	↓	
Ndufb7	Ndufb7	NADH dehydrogenase [ubiquinone] 1 beta subcomplex subunit 7	↓	
Ndufb10	Ndufb10	NADH dehydrogenase [ubiquinone] 1 beta subcomplex subunit 10	↓	
Ndufb11	Ndufb11	NADH dehydrogenase [ubiquinone] 1 beta subcomplex subunit 11	↓	
Ndufs2	Ndufs2	NADH dehydrogenase [ubiquinone] iron-sulfur protein 2	↓	
Ndufs4	Ndufs4	NADH dehydrogenase [ubiquinone] iron-sulfur protein 4	↓	
Ndufs5	Ndufs5	NADH dehydrogenase [ubiquinone] iron-sulfur protein 5	↓	
Ndufs6	Ndufs6	NADH dehydrogenase [ubiquinone] iron-sulfur protein 6	↓	
Ndufs7	Ndufs7	NADH dehydrogenase [ubiquinone] iron-sulfur protein 7	↓	
Ndufv1	Ndufv1	NADH dehydrogenase [ubiquinone] flavoprotein 1	↓	
Ndufv2	Ndufv2	NADH dehydrogenase [ubiquinone] flavoprotein 2	↓	

Uqcrfs1	Uqcrfs1	Ubiquinol-cytochrome c reductase, Rieske iron-sulfur polypeptide 1	↓	
Casp1	Casp1	Caspase-1	↑	Inflammation
Cd68	Cd68	Macrosialin	↑	
Mrc1	Mrc1	Macrophage mannose receptor 1	↑	
Itgam	Itgam	Integrin alpha-M	↑	
Stat2	Stat2	Signal transducer and activator of transcription 2	↑	
Rnasel	Rnasel	2-5A-dependent ribonuclease	↑	

References:

1. **Pablo JL, and Pitt GS**. FGF14 is a regulator of KCNQ2/3 channels. *Proc Natl Acad Sci U S A* 114: 154-159, 2017.
2. **Abbott GW, and Jepps TA**. Kcne4 Deletion Sex-Dependently Alters Vascular Reactivity. *J Vasc Res* 53: 138-148, 2016.
3. **Mani BK, Robakowski C, Brueggemann LI, Cribbs LL, Tripathi A, Majetschak M, and Byron KL**. Kv7.5 Potassium Channel Subunits Are the Primary Targets for PKA-Dependent Enhancement of Vascular Smooth Muscle Kv7 Currents. *Mol Pharmacol* 89: 323-334, 2016.
4. **Schuetz F, Kumar S, Poronnik P, and Adams DJ**. Regulation of the voltage-gated K(+) channels KCNQ2/3 and KCNQ3/5 by serum- and glucocorticoid-regulated kinase-1. *Am J Physiol Cell Physiol* 295: C73-80, 2008.
5. **Manville RW, Neverisky DL, and Abbott GW**. SMIT1 Modifies KCNQ Channel Function and Pharmacology by Physical Interaction with the Pore. *Biophys J* 113: 613-626, 2017.
6. **Barrese V, Stott JB, Baldwin SN, Mondejar-Parreno G, and Greenwood IA**. SMIT (Sodium-Myo-Inositol Transporter) 1 Regulates Arterial Contractility Through the Modulation of Vascular Kv7 Channels. *Arterioscler Thromb Vasc Biol* 40: 2468-2480, 2020.
7. **Yu Q, Xiao H, Jedrychowski MP, Schweppe DK, Navarrete-Perea J, Knott J, Rogers J, Chouchani ET, and Gygi SP**. Sample multiplexing for targeted pathway proteomics in aging mice. *Proc Natl Acad Sci U S A* 117: 9723-9732, 2020.
